# Cell subset prediction for blood genomic studies

**DOI:** 10.1186/1471-2105-12-258

**Published:** 2011-06-24

**Authors:** Christopher R Bolen, Mohamed Uduman, Steven H Kleinstein

**Affiliations:** 1 Interdepartmental Program in Computational Biology and Bioinformatics, Yale University, 300 George St. Suite 501, New Haven, Connecticut, 06511, USA; 2 Department of Pathology, Yale University School of Medicine, New Haven, Connecticut, USA

## Abstract

**Background:**

Genome-wide transcriptional profiling of patient blood samples offers a powerful tool to investigate underlying disease mechanisms and personalized treatment decisions. Most studies are based on analysis of total peripheral blood mononuclear cells (PBMCs), a mixed population. In this case, accuracy is inherently limited since cell subset-specific differential expression of gene signatures will be diluted by RNA from other cells. While using specific PBMC subsets for transcriptional profiling would improve our ability to extract knowledge from these data, it is rarely obvious which cell subset(s) will be the most informative.

**Results:**

We have developed a computational method (Subset Prediction from Enrichment Correlation, SPEC) to predict the cellular source for a pre-defined list of genes (i.e. a gene signature) using only data from total PBMCs. SPEC does not rely on the occurrence of cell subset-specific genes in the signature, but rather takes advantage of correlations with subset-specific genes across a set of samples. Validation using multiple experimental datasets demonstrates that SPEC can accurately identify the source of a gene signature as myeloid or lymphoid, as well as differentiate between B cells, T cells, NK cells and monocytes. Using SPEC, we predict that myeloid cells are the source of the interferon-therapy response gene signature associated with HCV patients who are non-responsive to standard therapy.

**Conclusions:**

SPEC is a powerful technique for blood genomic studies. It can help identify specific cell subsets that are important for understanding disease and therapy response. SPEC is widely applicable since only gene expression profiles from total PBMCs are required, and thus it can easily be used to mine the massive amount of existing microarray or RNA-seq data.

## 1 Background

Gene expression data from blood genomic studies are widely used for investigation of human disease [1]. Predictive gene signatures have been developed to carry out differential diagnosis of infectious diseases [2], identify specific disease states [3] and characterize the immune response to vaccination [4]. However, is some cases gene expression signatures from blood can be weakly expressed and highly variable [5]. The identification of these signatures is complicated by the fact that blood is a mixed tissue, composed of multiple cell subsets, so that differential expression profiles can reflect changes in cell subset proportions, changes in subset-specific gene expression or both. In cases where the relevant disease genes are expressed in a subset-specific manner, as has been shown for SLE [6], analyses based on mixed cell expression data are inherently limited since differential expression of genes in one cell subset (e.g., monocytes) will be diluted by RNA from other cells. Experimental studies that isolate specific cell subsets before expression profiling can provide important biological insight by demonstrating subset-specific gene expression as well as increased predictive signal [7]. Given the large number of potential cell subsets that can be defined, the ability to identify the most informative subset(s) to isolate would be a great aid to these studies.

**Figure 1:**
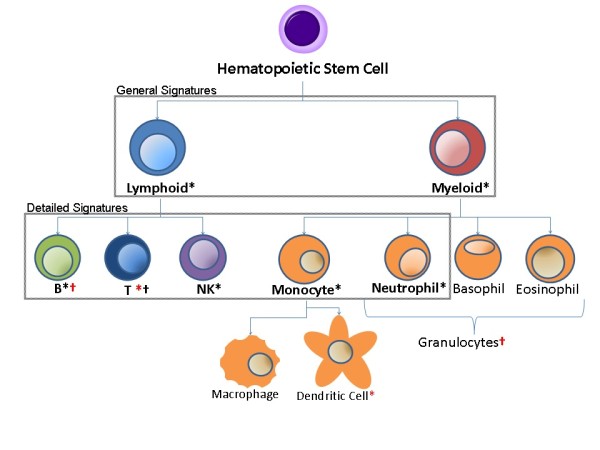
**Hematopoietic lineage tree**. Cell subset-specific gene expression signatures were obtained from Abbas et al. [12] (marked with *) and Palmer et al. [13] (marked with †). Only one signature for each subset was chosen for use in SPEC (black symbols). The remaining signatures (red symbols) were utilized as part of the independent subset validation (Table 3).

Most genome-wide expression studies are based on analysis of total peripheral blood mononuclear cells (PBMCs). PBMCs are composed of over a dozen cell subsets that are derived from a common progenitor in the bone marrow (Figure 1). These cells are commonly divided into myeloid and lymphoid cells. Myeloid cells include monocytes and their descendants, as well as granulocytes like neutrophils and basophils. Lymphoid cells are primarily composed of B cells, T cells and NK cells. Proportions of these cells can vary widely between individuals, but T cells and B cells together usually make up ~75% of PBMCs, while NK cells and Monocytes make up around 10-15% each. The remaining cell types, such as dendritic cells, are much more rare, and account for <1% of total PBMCs [8]. Neutrophils, which normally compose the majority of cells in a blood sample (40-80%), are normally excluded by the methods used to isolate PBMCs, but may account for up to 20% of a PBMC sample due to contamination [9].

Genome-wide expression measurements based on total PBMCs reflect both condition-specific gene expression as well as the proportion of different cell subsets in the sample. Microarray deconvolution methods have been developed to take advantage of this latter dependence in order to quantify the relative proportion of different cell subsets [10]. In this approach, the expression level of each gene is modeled as a linear function of the expression from each cell subset comprising the sample. Deconvolution thus depends on prior knowledge of quantitative expression levels for each subset. In cases where the subset proportions have been measured experimentally, methods have been developed to compute subset-specific differential-expression for individual genes [11]. However, in addition to requiring measurements that are not available for many existing datasets, these methods do not directly address the problem of predicting the cell subset source of a pre-defined gene signature (e.g., one that is externally-derived or biologically-motivated). In some cases, clues to the cellular source of a gene expression signature may be found in the signature itself. This would be true when the signature is enriched for genes known to be expressed in a particular cell type [1], and may occur when the proportion of cells in the blood changes in the condition of interest. For example, Berry et al. found that changes in T-cell related genes included in a transcriptional signature of active tuberculosis resulted from changes in T cell number in the blood [3]. In cases where the predictive signature does not include a significant number of cell subset-specific genes, the problem may be addressed experimentally through the isolation of specific cell subsets followed by transcriptional profiling [7].

However, it is often not obvious which cell subset(s) will be the most informative. The time and expense of isolating and analyzing all possibly relevant cell subsets provides the motivation for the method developed here: a computational approach to predict the most likely cell subset source of a gene expression signature. The development of this method was also motivated by the clinical problem of predicting the outcome of antiviral therapy in patients chronically infected with hepatitis C virus (HCV). In this case, gene expression changes that are predictive when measured in hepatocytes are significantly blunted in PBMCs, making it difficult to develop highly predictive models from this cell population [5]. The isolation of specific cell subsets prior to expression profiling may allow for the identification of predictive signatures based on a blood sample, with significant advantages for the patient.

We have developed a computational method to predict the most likely cellular source for a pre-defined gene expression signature using transcriptional profiling data from total PBMCs. The general approach we propose is to: (1) estimate the relative proportion of each PBMC subset from individual patients using genome-wide transcriptional profiling data, and (2) correlate these proportions with the gene expression signature that predicts therapy. We theorize that the PBMC subset proportion that correlates most closely with the predicted gene expression signature over a large set of patients is the most likely source of the signal. As a specific example, let's consider a study where we find that Interferon Stimulated Genes (ISGs) are up-regulated in a subset of patients that fail to respond to a particular therapy. Now, we focus on the non-responding patients. If ISG expression tends to be higher in patients where the proportion of dendritic cells (DCs) is predicted to be high, then we would predict that DCs are the most likely source of the observed gene expression signature. Note that there can be two interpretations of this result with respect to the original study. First, the DCs may always express ISGs, but the proportion of DCs in the blood is higher for non-responders. Second, the DCs undergo specific gene expression changes in non-responders which cause the level of ISG expression to increase in the blood sample. In either case, DCs are the relevant population to follow-up experimentally. Here, we develop this general idea into a computational method called SPEC (Subset Prediction from Enrichment Correlation), which we then validate using both healthy and disease data. While the development of SPEC was motivated by the problem of predicting the response to therapy in HCV patients, the method is generally applicable to link gene expression signatures with specific PBMC subsets. Since only gene expression profiles from total PBMCs are required, the technique can easily be used to mine the massive amount of existing microarray data.

## 2 Results

Subset Prediction from Enrichment Correlation (SPEC) is a method that takes advantage of the fluctuations of cell proportions in a mixed cell sample to find the most likely source for a gene expression signal. The general approach can be broken down into four steps:

1. Estimate the relative proportion of each PBMC subset (e.g., B cells, T cells, etc.) across a population. We propose to accomplish this by calculating the enrichment score (E[s, i] for PBMC subset **s **and individual **i**) associated with a set of genes expressed specifically in each subset **s**, called a subset signature (**subsetSignature[s]**). This is summarized by the following pseudo-code:

Code:

**N_P = **Number of individuals

**N_S **= Number of PBMC subsets

**exprData[i] = **gene expression profile for individual **i**

**subsetSignature[s] **= list of genes representing PBMC subset **s**

**querySignature = **a list of genes input by the user

for each (**i **in 1 to **N_P**)

for each (**s **in 1 to **N_S**)

**E**[s, i] = enrichment of **subsetSignature**[**s**] in **exprData**[**i**]

2. Calculate the enrichment score of the query signature for each individual **i **in the population:

for each (**i **in 1 to **N_P**)

**Q**[**i**] = enrichment of **querySignature **in **exprData**[**i**]

3. Determine the Pearson correlation (across the population) between the enrichment scores of the query signature and each PBMC subset **s**:

for each (**s **in 1 to **N_S**)

**C**[**s**] = correlation between **Q[i] **and **E**[**s**,**i**] across **i**

4. Predict the subset with the highest correlation to the query signature:

Predicted subset = **s **that gives rise to the max(**C**[**s**])

**Figure 2:**
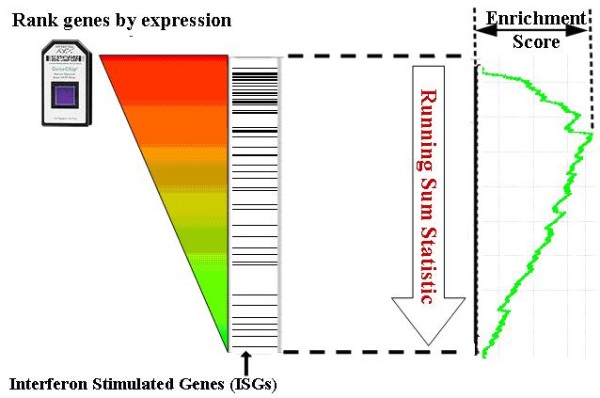
**The enrichment score calculation**. Genes are ranked based on their normalized expression levels and a running sum statistic is calculated from an a priori defined gene signature (the query or cell subset-specific signature in our framework). The enrichment score is defined as the maximum deviation from zero of the running sum. Additional details can be found in methods.

The calculation of enrichment scores is explained in Methods and Figure 2.

### 2.1 Estimating relative PBMC subset proportions using enrichment scores

The first step in our proposed method is to estimate the relative proportion of each PBMC subset. While a number of methods exist that can estimate these values, we chose to use enrichment scores calculated from cell subset-specific gene signatures for our initial implementation of SPEC due to its relative simplicity. As described in the methods, the enrichment score calculates the extent to which a gene signature is concentrated among the most highly expressed genes in a single genome-wide expression experiment. The cell subset-specific signatures are defined as groups of genes which have been found to be up-regulated in only a specific subset of cells in a mixed cell population. The resulting lists of up-regulated genes represent cell markers for that blood cell type, and include well known cell surface markers such as CD3 for T cells and CD19 for B cells. Lists of genes describing specific PBMC subsets have previously been defined in [12] and in [13]. Here, we choose to use signatures representing the B cell, T cell, NK cell, and monocyte subsets, We also included a signature for neutrophils despite the fact that they are not considered PBMCs, since neutrophil contamination can account for up to 20% of a PBMC sample [9]. In addition, we also use a more general set of signatures for the lymphoid and myeloid subsets (Figure 1). The lymphoid and myeloid signatures are composed of genes which are expressed in more than one cell type from those lineages, thus these subsets should vary according to the sum of all the cells in that lineage. We run SPEC separately on the detailed (B cell, T cell, NK cell, neutrophil and monocyte) and general (lymphoid and myeloid) signatures, as described below.

**Figure 3:**
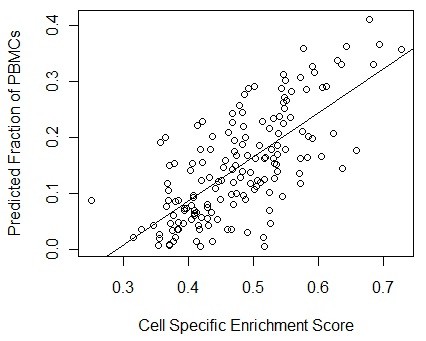
**Subset-specific enrichment scores correlate with predicted cell fraction by microarray deconvolution**. The fraction of NK cells was estimated from the set of healthy PBMC gene expression profiles using microarray deconvolution (y axis), and enrichment scores based on the NK cell subset signature (x axis). Each point is an individual patient (r = 0.71).

We hypothesize that the enrichment scores of the subset-specific gene signatures will be related to the relative cell subset proportions across patients. Although microarray deconvolution can be used to accomplish this task [10], these methods require quantitative gene expression levels for each subset to be estimated, which is not always available. In addition, it is important to be clear that we do not require the enrichment scores of different cell subsets to predict their proportions in a single individual (as attempted by microarray deconvolution). Rather, we propose that the enrichment score for a single cell subset will vary along with the relative size of the cell subset across patients. For example, if subject B has a higher fraction of NK cells than subject A then the NK signature enrichment score should also be higher, but both of these enrichment scores may be higher than the T cell signature enrichment scores even if NK cells constitute a lower fraction of cells in both subjects. In order to validate this enrichment score approach, we compared it with predictions from microarray deconvolution [10] using data from healthy subjects where we expect deconvolution should perform relatively well. We predicted the relative proportions of cell subsets for 161 healthy gene expression profiles using both microarray deconvolution and the cell signature enrichment scores. Figure 3 is an example showing the correlation between the NK cell signature enrichment score and the predicted NK cell fraction estimated using deconvolution. The correlation of 0.71 implies that the enrichment score is positively varying with the relative proportion of NK cells in these healthy PBMC samples. We found similarly high correlations for B cells, neutrophils and monocytes (0.65, 0.70 and 0.87 respectively). In contrast, T cells (r = 0.21) had a much weaker correlation, perhaps reflecting the quality of this particular gene signature. Overall, these results suggest that enrichment scores can be used to track the fluctuations in the levels of each cell type in a mixed cell expression profile (i.e., from observations on total PMBCs).

### 2.2 Validation of SPEC using split subset signatures

As an initial validation of SPEC, we sought to predict the source of a gene expression signature where the correct cell subset is known. This can be done by using our pre-defined cell subset expression signatures. For example, consider the B cell signature defined in [12]. The genes that are part of this signature can be split into two disjoint sets. One of these sets can be labeled as the cell subset signature and used to estimate the changes in the B cell population, while the other can be retained as the query gene signature being tested. A successful method would be able to identify this query signature as coming from B cells or from lymphoid cells. When running SPEC, we separately analyze the set of detailed signatures (B cell, T cell, NK cell, neutrophil and monocyte) and general signatures (lymphoid and myeloid), which represent cell types that are a superset of those covered by detailed signatures (Figure 1).

For each test, one of the subset signatures was randomly divided into two halves, with one half used as the query signature and the other half retained as the subset signature. This was repeated 200 times for each of the PBMC subset signatures. In order to determine the significance of the correlation value for each run we used a Monte Carlo permutation test (see Methods). The following two subsections describe the performance of SPEC on healthy and disease data.

#### 2.2.1 Performance using healthy data

**Figure 4:**
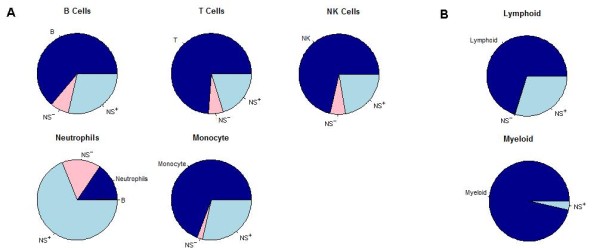
**Performance of SPEC using split subset-specific signatures on gene expression profiles from healthy subjects**. Each of the cell subset-specific signatures used in SPEC (individual pie charts) was randomly split 200 times, with half of the genes used as the query signature and the other half retained as the cell subset signature. SPEC was considered to be correct if it linked the query to the subset from which it was derived (shades of blue) and incorrect otherwise (shades of red). Significance cutoffs were calculated for each run to determine whether the link between query and subset was statistically significant (p < 0.05, dark blue/red) or not (p > = 0.05, light blue/pink). Predictions that were incorrect and statistically significant are separated and labeled with the predicted subset. Predictions that did not reach the level of statistical significance are labeled NS+ and NS- to indicate correct and incorrect predictions, respectively. SPEC was applied separately to predict the source of query signatures coming from (A) B cells, T cells, NK cells, neutrophils or monocytes, and (B) lymphoid or myeloid cells.

A set of 161 PBMC gene expression profiles from healthy subjects was collected from six published studies [14-18] (see Methods for details). Figure 4 summarizes the results of running SPEC on these data using the split subset signature validation test described above. Results were binned into significant vs. non-significant groups using a p-value cutoff of 0.05. Overall, SPEC very accurately predicts the source of the query signal for all split signatures. Nearly all significant predictions correctly linked the query to its proper subset and, even including the non-significant results, there were relatively few false positives. For most of the subset signatures, approximately 75% of the runs were significant and correct. As an important negative control, we found that most results from the neutrophil subset did not reach statistical significance reflecting that fact that granulocytes (including neutrophils) are mostly absent in PBMCs [9,13]. Interestingly, a large portion of the non-significant results were still correct, most likely because contamination from neutrophils still provides a small amount of signal in the PBMC samples.

#### 2.2.2 Performance in patients with disease

The previous validation experiments show that SPEC works for PBMC expression profiles from healthy subjects, but it is possible that the altered expression patterns in disease patients could interfere with the accuracy of SPEC. To verify that SPEC works in a disease setting, we repeated the validation steps described in section 2.2.1 using PBMC expression data from 94 patients with HCV [5,19,20] (Table 1), as well as 54 patients with Systemic Lupus Erythematosus (SLE) [1] (Table 2). For the HCV data, nearly all significant results correctly predicted the source of the query signature, although overall there were fewer results that were considered significant than in the healthy data. The SLE data, on the other hand, actually performed better on the B and T signatures with 100% of the runs being correct and significant but, as expected, SPEC was often unable to predict the correct source of a neutrophil signature. The monocyte signature also was incorrectly associated with neutrophils about half the time, suggesting that performance could be improved by removing cell subset signatures that should be absent from a sample. We conclude that, despite some quantitative differences in the performance measures compared with data from healthy subjects, SPEC is not overly affected by potential differential gene expression induced in SLE or HCV, and can be effectively applied to data from individuals in a disease setting.

**Table 1:** Performance of SPEC for linking split subset signatures using data from HCV patients

	B	T	NK	Neutrophil	Monocyte
B	**37 (69)**	0 (30)	0 (1)	0 (0)	0 (0)

T	0 (8)	**14 (91)**	0 (1)	0 (0)	0 (0)

NK	0 (9)	0 (13)	**20 (56)**	0 (1)	0 (23)

Neutrophil	0 (2)	0 (1)	0 (1)	**22 (41)**	3 (56)

Monocyte	0 (0)	0 (0)	0 (0)	1 (35)	**37 (66)**

Values are the percentage of time the query signature (rows) had the highest correlation with each subset signature (columns). Values are of the form: % significant (total %). Cells marked in bold indicate the highest value for each query signature. Rows may not add up to 100% due to rounding.

**Table 2:** Performance of SPEC for linking split subset signatures using data from SLE patients

	B	T	NK	Neutrophil	Monocyte
B	**100 (100)**	0 (0)	0 (0)	0 (0)	0 (0)

T	0 (0)	**100 (100)**	0 (0)	0 (0)	0 (0)

NK	0 (0)	1 (11)	**67 (88)**	0 (2)	0 (0)

Neutrophil	0 (0)	0 (0)	0 (0)	31 (33)	**66 (68)**

Monocyte	0 (0)	0 (0)	0 (0)	42 (47)	**47 (53)**

Values are the percentage of time the query signature (rows) had the highest correlation with each subset signature (columns). Values are of the form: % significant (total %). Cells marked in bold indicate the highest value for each query signature. Rows may not add up to 100% due to rounding.

### 2.3 Validation of SPEC using independently-derived signatures

#### 2.3.1 Performance using independent subset signatures

An alternative method for validating SPEC is to use signatures for the same cell subset that were generated from independent sources. To carry this out, we rely on studies by Abbas et al. [12] and Palmer et al. [13], which both defined signatures for B cells and T cells, as well as a signature for either granulocytes or neutrophils (neutrophils are a type of granulocyte). While these signatures have surprisingly little overlap with each other, we nevertheless removed the small number of common genes from the query signatures before running SPEC to avoid biasing the performance measurements. As shown in Table 3, SPEC correctly predicts the source of the B cell signature, and the granulocyte signature was appropriately identified with the neutrophil signature. The granulocyte signature is also correctly predicted as coming from the myeloid cell subset (see Figure 1). The T cell signature on the other hand does not show a significant correlation with any subset signature. This is likely caused by problems with one of the T cell signatures, as we already found that this signature had the poorest performance in the validation of enrichment scores using deconvolution values (see section 2.1).

**Table 3:** Linking independently-derived cell-subset signatures

	B Cells	T Cells	Granulocyte
B Cells	0.82	-0.29	0.16

T Cells	-0.08	-0.07	0.13

NK Cells	0.15	-0.29	0.27

Neutrophils	0.41	-0.56	0.80

Monocyte	-0.22	0.09	0.40

			

Lymphocyte	0.00	0.22	-0.39

Myeloid Cells	0.15	-0.39	0.86

Values indicate the correlation between the enrichment scores calculated by the Primary signatures (rows) and the independently derived signatures (columns). Highlighted cells are correlations with p < 0.05

#### 2.3.2 Performance using experimentally-derived subset-specific disease signatures

**Figure 5:**
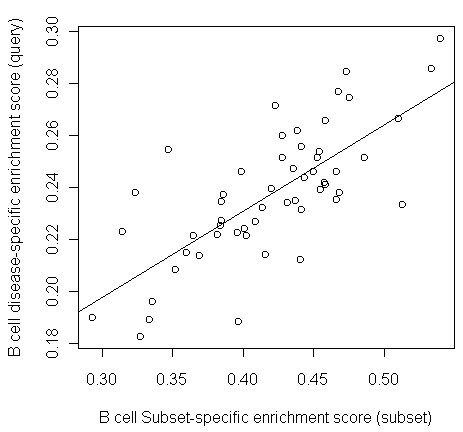
**The experimentally-derived B cell-specific disease signature is significantly correlated with the B cell subset-specific signature across a population of SLE patients**. Enrichment scores were calculated for each patient (points) using either the B cell subset-specific signature from SPEC (x axis) or the B cell-specific disease signature produced using data from experimentally purified B cells (see methods). The sample correlation between these enrichment scores is r = 0.73, which is significantly higher than the correlation of the subset signature with randomly generated signatures (p < 0.05).

Our final validation approach involved testing the ability of SPEC to accurately predict the source of experimentally-determined query signatures derived from purified cell subsets from SLE patients. In this case transcriptional profiling data was measured specifically from purified B cells, T cells and myeloid cells [6]. We defined cell subset-signatures as the set of genes that were up-regulated in SLE patients (compared with healthy controls) and that did not overlap with differentially-expressed genes from other cell subsets (see Methods). SLE signatures for the three cell subsets were used as query signatures and the subset source was predicted with SPEC. As expected, query signatures created using a particular cell subset were linked by SPEC to the correct subset using only the gene expression data from total PBMCs. The B cell specific disease signature has the highest correlation with the B cell subset signature (r = 0.73, p < 0.001, Figure 5). Similarly, the T-cell specific disease signature has the highest correlation with the T cell subset signature (r = 0.72, p < 0.001). The myeloid signature had high correlation with myeloid cells in the myeloid vs. lymphoid comparison (r = 0.75, p < 0.0001), and also had the highest correlations with all of the myeloid-derived subset signatures in the more detailed comparisons. Thus, SPEC is capable of predicting the source of a disease-associated subset-specific gene expression pattern using only gene expression from total PBMCs.

#### 2.3.3 Effect of query signature size on accuracy

**Figure 6:**
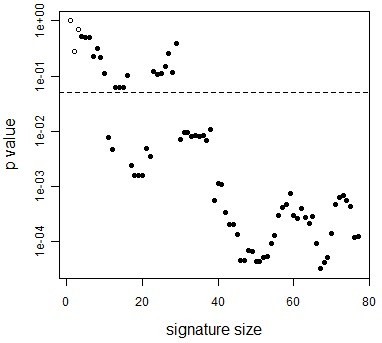
**Effect of query signature size on SPEC accuracy**. The B cell-specific disease signature was trimmed to the size indicated on the x-axis by removing the least differentially-expressed genes. SPEC was then applied to the SLE dataset in order to identify the source of this query signature using the detailed cell subsets. Predictions were considered correct (filled circles) when the most likely subset was determined as B cells, and incorrect otherwise (empty circles). The dashed line indicates a p-value cutoff of 0.05.

The size of the SLE subset-specific signatures used in the previous section was fixed as the top-100 up-regulated genes. We used a fixed cutoff so that we could easily compare between results on the different subsets. In practice, drawing a cutoff or choosing the number of genes to include in a gene expression signature can be difficult, and the number of genes that reach statistical significance for differential expression may be small. Thus, we sought to investigate the effect of query gene signature size on the accuracy of SPEC predictions using the SLE disease signatures from section 2.3.2. The size of the query signature was successively decreased, and SPEC was applied to each of the query signatures (Figure 6). As expected, p-values generally increased with smaller signatures and, in this case, the results were no longer significant when gene signatures contained less than 15 genes. Interestingly, this is also the minimum number of genes recommended for calculating robust enrichment scores in GSEA [21]. All significant results were correct and, even with the smaller subset sizes that failed to reach statistical significance, SPEC often predicted the correct subset source. These results demonstrate the importance of robust subset signatures in the application of SPEC, but also highlight the fact that SPEC can be effective even when using relatively small query gene sets.

### 2.4 Case study: identifying the ISG signature source for predicting therapy response in chronic HCV patients

The current standard therapy for chronic HCV consists of pegylated (PEG) Interferon (IFN)-α and ribavirin. However, this treatment is costly, poorly tolerated due to adverse side effects, and ineffective in many patients [22]. The majority of HCV infections in the United States are with genotype 1, which responds poorly to therapy (40% response rate) [23]. Methods to identify patients who are likely to be responsive to therapy would be a great help in clinical decision making. In addition, the ability to predict non-responsiveness could allow alternate treatments to be explored. Previous studies have found that patients who fail to achieve a sustained virologic response (SVR) after treatment display high baseline levels of IFN-stimulated gene (ISG) expression in hepatocytes prior to the initiation of therapy [24,25], and the expression of these ISGs is not increased by IFN treatment. However, gene expression changes in total PBMCs in HCV patients appear blunted compared with looking directly at hepatocytes, making it difficult to develop highly predictive models from this cell population [5,25]. Because there are significant advantages to using PBMCs for interrogating disease processes and performing clinical tests, it is important to investigate ways to improve the predictive power of PBMC gene expression profiling. We hypothesize that the accuracy of existing models based on PBMCs is limited because differential expression of ISGs is likely to be cell type-specific, and thus their signal will be diluted in total PBMC by RNA from other cells. This logic applies to non-ISG signatures as well. Therefore, using specific PBMC subsets for transcriptional profiling should improve our ability to extract knowledge from these data. Unfortunately, it is not obvious which cell subset(s) are the most informative, so we have applied SPEC to predict the most likely cellular source for predictive signatures in HCV.

We first generated a list of genes that were significantly up-regulated in patients classified as non-responders (compared with responders) based on gene expression profiling data from total PBMCs in 16 chronic HCV patients generated by Sarasin-Filipowicz et al. [5]. Using this gene list as the query signature, SPEC was then applied to the PBMC expression profiles from only the 6 non-responders in this study (see Methods) in order to predict the cellular source of the up-regulated genes. SPEC identified the myeloid subset (r = 0.74, p < 0.05), as the most likely source for this query signature. None of the associations with the detailed signatures reached statistical significance. This could be due in part to the relatively small number of samples used in the analysis. It is also possible that the therapy-response signature arises from multiple cell types in the myeloid family, or possibly from Dendritic cells (DCs), which were not included in our subsets for reasons previously described. Overall, these results suggest that gene expression analysis on the myeloid subset should improve our ability to predict therapy response in HCV patients.

## 3 Discussion

Blood is a mixture of many different cell types. The ability to associate a predictive gene expression signature with a specific cell subset would allow better classifiers to be developed through the experimental isolation of the most relevant cells. In addition, knowledge of which cells are being affected would provide important biological insights. No other computational methods exist to directly address this problem. In this study we developed a computational method, Subset Prediction from Enrichment Correlation (SPEC), to predict the most likely cellular source of a predictive gene expression signature. Our method requires only gene expression data from total PBMCs, and does not depend on knowing the subset proportions in the blood sample. SPEC uses enrichment scores to estimate the strength of gene expression signatures across individuals in a population. The PBMC subset signature showing the highest correlation with the query gene signature is predicted to be the source. We show that SPEC has good performance on both healthy and disease data, and correctly predicts the source of experimentally-derived subset-specific genes in SLE patients.

As a case study, we applied SPEC to transcriptional profiling data from chronic HCV patients in order to predict the potential source of a gene expression signature associated with non-responsiveness to standard therapy. Using SPEC, we predict that a stronger signal for non-responsiveness will be apparent when focusing on the myeloid subset. We hope that looking at the cell subpopulations separately will allow us to develop a blood test that can predict the clinical outcome of antiviral therapy prior to treatment.

The quality of cell subset gene expression signatures is critical to the performance of SPEC. It is clear from our results (e.g., sections 2.2.2 and 2.3.2) that some signatures are simply better than others. In this work, we use gene expression signatures for B cells, T cells, NK cells, neutrophils and monocytes available from [12] and [13]. We decided to exclude the DC subset signatures from [12] since we found that it did not work well within the SPEC framework. We speculate that this is due to the fact that DCs are such a small component of the total PBMCs (less than 1% on average [8]). More work is needed to define the qualities of a "good" signature for the SPEC framework. Other groups have proposed additional signatures that we plan to evaluate [26], with the long-term goal of defining a set of non-overlapping signatures for a detailed set of PBMC subsets. As an alternative to applying SPEC simultaneously to all available subsets, it is possible SPEC may be applied in a hierarchical manner: first predicting whether the query comes from the lymphoid vs. myeloid subsets then, if lymphoid, predict B cell vs. T cell vs. NK cell then, if B cell, predict naïve B cell vs. memory B cell vs... and so on.

An additional area for future investigation concerns alternatives to the enrichment score, which is currently used for estimating both the relative cell subset proportions and expression of the query signature. One possibility that we have already investigated is using microarray deconvolution, described in [10] and in section 2.1, to measure cell subset fractions. However, we found this method actually reduced the accuracy of SPEC (data not shown). Future work could explore other methods for estimating cell proportions from gene expression data [27,28]. One thing to keep in mind is that SPEC does not require estimating the absolute proportion of all cell types within a single individual as provided by deconvolution methods, but rather assumes that we can estimate the relative proportion for each individual cell subset across a population. By trying to solve a harder problem, deconvolution methods may actually introduce more error compared with enrichment scores. Another advantage of enrichment scores is that they are likely to be less dependent on sample source (e.g., PBMCs vs. whole blood).

## 4 Conclusion

This work describes the implementation and validation of a computational methodology to support blood genomics studies with general applicability to basic and translational research. SPEC predicts the cellular source of predictive gene expression signatures. When applied to data from chronic HCV patients, SPEC predicts that focusing on myeloid cells may enrich for an interferon signature that has been observed in hepatocytes from patients that do not respond to standard therapy. The approach requires only transcriptional profiling data from total PBMCs, allowing wide applicability, and should improve the predictive power of blood gene expression profiling studies by allowing experiments to focus on the most informative cell subsets. It is also easy to envision how this framework could be combined with classification methods to define predictive signatures that are more likely to be cell subset-specific, and therefore amenable to experimental signal enrichment through sorting.

## 5 Methods

### Microarray data and normalization

Raw Affymetrix microarray data were downloaded from GEO. Transcriptional profiles from PBMS were obtained for healthy controls (GSE11057, GSE11761, GSE14642, GSE15645, GSE7753, GSE8507), SLE patients (GSE11908), and chronic HCV patients (GSE7123, GSE11342, GSE11190). Cell specific transcriptional profiles for B cells, T cells and myeloid cells from SLE patients were obtained from GSE10325. These data were normalized with GCRMA [29] using the BioConductor software package in R [30].

### Generation of SLE cell specific disease signatures

Differentially-expressed genes were determined for each cell subset (B, T and myeloid cells) by comparing SLE and healthy individuals using the LIMMA package in BioConductor [31]. Genes were separated into groups of upregulated and downregulated genes, ranked by FDR, and the top 100 genes were pulled out. Cell specific disease signatures were created by using only the genes unique to each signature.

### Generation of HCV response signature

Gene expression data was obtained from the study by Sarasin-Filipowicz et al. [5]. Differentially-expressed genes were determined by comparing non-responders vs. responders using the LIMMA package in BioConductor [31]. Significance was defined using a false discovery rate cutoff of 0.1 using the method of Benjamini and Hochberg.

### Enrichment score calculation

The enrichment score is calculated as described in [21] with p = 1 and weighted by the gene rank. Genes are ranked based on their expression values from a single transcriptional profile (highest to lowest expression). The enrichment score is the maximum deviation from zero of a running sum statistic which is calculated by walking down the ranked list of genes. The sum is incremented whenever a gene in the signature is encountered and decremented if the gene is not in the signature. Increments and decrements are weighted so the statistic sums to 0 over all the genes.

### Microarray deconvolution

Deconvolution was performed to estimate the fraction of each cell subset from a single PBMC transcriptional profile using the methods described in [10]. The cell subset expression data used for deconvolution was downloaded from the supplementary material. The predicted cell proportions of related subsets were added together to generate the final cell fraction (e.g., CD4^+ ^+ CD8^+ ^+ T cell = Proportion of T cells).

### Monte Carlo permutation test for P value estimation

To calculate a P value for the maximum correlation coefficient found by SPEC, we estimated the distribution of these values under the null hypothesis by randomly permuting the assignment of genes on the microarray to the query signature, while maintaining the size of the query signature. We then ran SPEC to determine the maximum correlation between the enrichment scores for these new queries and each subset signature. This process was repeated 1000 times to generate a distribution of maximum correlations. P-values were then estimated by fitting a normal distribution to these data and calculating the cumulative density for each SPEC result.

## Implementation and Availability

SPEC was implemented in the R statistical programming language, and is available at http://clip.med.yale.edu/SPEC. Additional requests can be made by contacting CRB or SHK.

## Authors' contributions

CRB helped design the study, programmed the SPEC algorithm and carried out the validation and analysis. MU helped with programming and the validation using the SLE data. SHK conceived of the study and participated in its design and coordination. CRB and SHK drafted the manuscript. All authors have read and approved the final manuscript.

## Competing interests

The authors declare that they have no competing interests.

## References

[B1] Chaussabel D A modular analysis framework for blood genomics studies: application to systemic lupus erythematosus Immunity 2008 29 1 150 64 10.1016/j.immuni.2008.05.012 18631455 PMC2727981

[B2] Ramilo O Gene expression patterns in blood leukocytes discriminate patients with acute infections Blood 2007 109 5 2066 77 10.1182/blood-2006-02-002477 17105821 PMC1801073

[B3] Berry MP An interferon-inducible neutrophil-driven blood transcriptional signature in human tuberculosis Nature 2010 466 7309 973 7 10.1038/nature09247 20725040 PMC3492754

[B4] Querec TD Systems biology approach predicts immunogenicity of the yellow fever vaccine in humans Nat Immunol 2009 10 1 116 25 10.1038/ni.1688 19029902 PMC4049462

[B5] Sarasin-Filipowicz M Interferon signaling and treatment outcome in chronic hepatitis C Proc Natl Acad Sci USA 2008 105 19 7034 9 10.1073/pnas.0707882105 18467494 PMC2383932

[B6] Hutcheson J Combined deficiency of proapoptotic regulators Bim and Fas results in the early onset of systemic autoimmunity Immunity 2008 28 2 206 17 10.1016/j.immuni.2007.12.015 18275831

[B7] Grigoryev YA Deconvoluting post-transplant immunity: cell subset-specific mapping reveals pathways for activation and expansion of memory T, monocytes and B cells PLoS One 5 10 e13358 10.1371/journal.pone.0013358 PMC2954794 20976225

[B8] Autissier P Evaluation of a 12-color flow cytometry panel to study lymphocyte, monocyte, and dendritic cell subsets in humans Cytometry A 77 5 410 9 10.1002/cyto.a.20859 PMC11742174 20099249

[B9] Schlenke P Evaluation of a novel mononuclear cell isolation procedure for serological HLA typing Clin Diagn Lab Immunol 1998 5 6 808 13 9801339 10.1128/cdli.5.6.808-813.1998 PMC96206

[B10] Abbas AR Deconvolution of blood microarray data identifies cellular activation patterns in systemic lupus erythematosus PLoS One 2009 4 7 e6098 10.1371/journal.pone.0006098 19568420 PMC2699551

[B11] Shen-Orr SS Cell type-specific gene expression differences in complex tissues Nat Methods 2010 7 4 287 9 10.1038/nmeth.1439 20208531 PMC3699332

[B12] Abbas AR Immune response in silico (IRIS): immune-specific genes identified from a compendium of microarray expression data Genes Immun 2005 6 4 319 31 10.1038/sj.gene.6364173 15789058

[B13] Palmer C Cell-type specific gene expression profiles of leukocytes in human peripheral blood BMC Genomics 2006 7 115 10.1186/1471-2164-7-115 16704732 PMC1479811

[B14] Fall N Gene expression profiling of peripheral blood from patients with untreated new-onset systemic juvenile idiopathic arthritis reveals molecular heterogeneity that may predict macrophage activation syndrome Arthritis Rheum 2007 56 11 3793 804 10.1002/art.22981 17968951

[B15] Holland SM STAT3 mutations in the hyper-IgE syndrome N Engl J Med 2007 357 16 1608 19 10.1056/NEJMoa073687 17881745

[B16] Knowlton N The meaning of clinical remission in polyarticular juvenile idiopathic arthritis: gene expression profiling in peripheral blood mononuclear cells identifies distinct disease states Arthritis Rheum 2009 60 3 892 900 10.1002/art.24298 19248118 PMC2758237

[B17] Radom-Aizik S Brief bout of exercise alters gene expression in peripheral blood mononuclear cells of early- and late-pubertal males Pediatr Res 2009 65 4 447 52 10.1203/PDR.0b013e3181993473 19127215 PMC4065861

[B18] Radom-Aizik S A brief bout of exercise alters gene expression and distinct gene pathways in peripheral blood mononuclear cells of early- and late-pubertal females J Appl Physiol 2009 107 1 168 75 10.1152/japplphysiol.00121.2009 19407257 PMC2711785

[B19] Taylor MW Changes in Gene Expression during Pegylated Interferon and Ribavirin Therapy of Chronic Hepatitis C Virus Distinguish Responders from Nonresponders to Antiviral Therapy J Virol 2007 81 7 3391 3401 http://jvi.asm.org/cgi/content/abstract/81/7/3391%U 10.1128/JVI.02640-06 17267482 PMC1866036

[B20] Taylor M Cyclic changes in gene expression induced by Peg-interferon alfa-2b plus ribavirin in peripheral blood monocytes (PBMC) of hepatitis C patients during the first 10 weeks of treatment Journal of Translational Medicine 2008 6 1 10.1186/1479-5876-6-66 PMC2613871 18986530

[B21] Subramanian A Gene set enrichment analysis: a knowledge-based approach for interpreting genome-wide expression profiles Proc Natl Acad Sci USA 2005 102 43 15545 50 10.1073/pnas.0506580102 16199517 PMC1239896

[B22] Poynard T Viral hepatitis C Lancet 2003 362 9401 2095 100 10.1016/S0140-6736(03)15109-4 14697814

[B23] Zeuzem S Interferon-based therapy for chronic hepatitis C: current and future perspectives Nat Clin Pract Gastroenterol Hepatol 2008 5 11 610 22 18838975 10.1038/ncpgasthep1274

[B24] Asselah T Gene Expression and Hepatitis C Virus Infection Gut 2009 10.1136/gut.2008.166348 PMC2673514 19074178

[B25] He XS Global transcriptional response to interferon is a determinant of HCV treatment outcome and is modified by race Hepatology 2006 44 2 352 9 10.1002/hep.21267 16871572

[B26] Watkins NA A HaemAtlas: characterizing gene expression in differentiated human blood cells Blood 2009 113 19 e1 9 10.1182/blood-2008-06-162958 19228925 PMC2680378

[B27] Clarke J Seo P Clarke B Statistical expression deconvolution from mixed tissue samples Bioinformatics 26 8 1043 9 10.1093/bioinformatics/btq097 PMC2853690 20202973

[B28] Erkkila T Probabilistic analysis of gene expression measurements from heterogeneous tissues Bioinformatics 26 20 2571 7 10.1093/bioinformatics/btq406 PMC2951082 20631160

[B29] Wu Z Irizarry RA Preprocessing of oligonucleotide array data Nat Biotechnol 2004 22 6 656 8 author reply 658. 15175677 10.1038/nbt0604-656b

[B30] Gentleman RC Bioconductor: open software development for computational biology and bioinformatics Genome Biol 2004 5 10 R80 10.1186/gb-2004-5-10-r80 15461798 PMC545600

[B31] Smyth GK Linear models and empirical bayes methods for assessing differential expression in microarray experiments Stat Appl Genet Mol Biol 2004 3 1 Article3 16646809 10.2202/1544-6115.1027

